# Complete Distal Rupture of the Rectus Femoris in an Elite Football Player: A Non-operative Treatment

**DOI:** 10.7759/cureus.45494

**Published:** 2023-09-18

**Authors:** Alexandre Fernandes, Julio Pinto, Pedro Cunha, Carlos Duarte, Alexandre Estaca, Tiago Pereira, Mónica Bettencourt, Isabel Candelária, Miguel Reis E Silva

**Affiliations:** 1 Health and Performance Unit, Casa Pia Atlético Clube - Futebol Sduq, Lisbon, PRT; 2 Physical Medicine and Rehabilitation, Hospital de Cascais, Lisbon, PRT; 3 Radiology, Clínica Lambert, Lisbon, PRT; 4 Sports Medicine, Myalgia Clinic, Lisbon, PRT

**Keywords:** return-to-performance, sports rehabilitation, return-to-play, elite football, muscle injury

## Abstract

Although muscle injuries represent the most frequent injury in professional football, isolated complete distal ruptures of the rectus femoris (RF) muscle are rare, and there is no consensus on their treatment and return to play (RTP). In this article, we report a clinical case of successful non-surgical management of an RF grade 4c muscle injury in a professional football player, in which the athlete was able to RTP 21 weeks after the injury, had no re-injury >1 year after RTP, and is playing at an elite level in the Portuguese Football First League.

## Introduction

Top-level football players typically sustain two injuries per season on average, leading to a combined 50 injuries within a squad of 25 players [1]. According to a report from the international insurance company Howden Group Holdings, during the 2021-2022 season, the English Premier League experienced the most injuries among the top-five European leagues (i.e., Spanish LaLiga, Italian Serie A, German Bundesliga, and French Ligue 1), with the league spending a total of 219.64 million euros in salaries to injured players during the season [2]. Fast recovery can have significant financial and strategic benefits for the player, the team, and the club [1,3]. Statistically, teams that reduce time loss from injuries often achieve greater league success [1,3,4].

In professional football, muscle injuries are the most common problem for players and medical and technical staff, accounting for substantial time lost in competition [4-6]. As specified by the UEFA Elite Club Injury Study Report 2019/20, the largest running injury surveillance program in football, muscle injuries represented 51% of all injuries in the participating clubs during the 2019/2020 season [7]. Of all muscle injuries, 57% were of the thigh, and 14% were considered severe, in which return to play (RTP) took more than 28 days [7]. Despite the current advances and knowledge in muscle biology, the incidence of muscle injuries in men’s professional football has not decreased, neither in training nor in matches [8].

Quadriceps muscle injuries are common in athletes, with the rectus femoris (RF) being the most frequently injured quadriceps muscle [9]. However, isolated complete distal ruptures of the RF are rare, and data on its management remains scarce [10,11]. The most common location of muscle injuries is at the muscle-tendon junction, and there is evidence that a muscle injury with tendon involvement is associated with a worse prognosis [6].

It is of utmost clinical importance to also understand the architecture of the knee extensor mechanism [12,13]. The extensor mechanism is a mechanical advantage that provides the critical function of knee extension, essential for normal gait and consequently sports practice [14,15]. The distal quadriceps tendon has a complex but constant architecture of three layers, in which the superficial layer is formed by the tendon of the RF [12,13,15,16].

Acute knee extensor mechanism injuries can potentially drastically alter an athlete’s career [15]. Despite its importance, there is limited evidence available in the literature for this kind of injury in elite professional-level sports, with most of the clinical cases published involving a complete rupture of the quadriceps tendon, in which surgical repair is the established gold standard [11,16,17].

In this article, we outline the conservative management and RTP process after a complete distal rupture of the RF in an elite football player.

## Case presentation

A 30-year-old male, professional football player, an ambidextrous midfielder, sustained a pre-season in-game injury on his support limb in a deceleration movement when receiving the ball. The athlete felt a slight pain in the anterior aspect of his left thigh and heard an intense pop, and was subsequently removed from the field.

**Figure 1:**
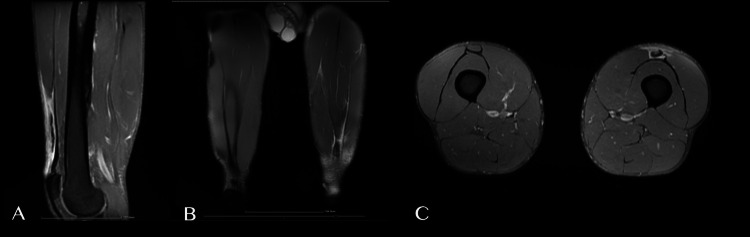
MRI T2-weighted sequence. (A) Sagittal plane. (B) Coronal plane. (C) Axial plane The images are compatible with a BAMIC grade 4c distal RF injury of the left thigh

On examination, we noted a visible and palpable deformity distally in the left quadriceps, with tenderness on palpation. The extensor mechanism was not affected, and the athlete could walk without any major complaints or limitations. We performed an ultrasound and an MRI scan, showing a complete distal rupture of the RF with tendon retraction, 34 mm away from the superior pole of the patella, compatible with a British Athletics Muscle Injury Classification (BAMIC) grade 4c RF injury, as seen in Figure 1.

The BAMIC proposed by Pollock et al. is a muscle injury classification system that grades injuries according to severity (grade 0-4) and anatomical site with an additional suffix ("a": myofascial; "b": musculotendinous junction, or "c": intramuscular tendon) based on MRI features within muscle or tendon. According to BAMIC, a grade 4 injury is a complete rupture to either the muscle (grade 4) or tendon (grade 4c) [6].

After discussing with the orthopedic surgeon consultant of the club and receiving a detailed explanation about the advantages and disadvantages of each option, the athlete chose conservative management. We designed a specific and functional rehabilitation program based on symptoms and muscle biology. The athlete used crutch ambulation for five days, without knee orthosis, with progressive loading.

The rehabilitation program was divided according to the three chronobiological phases of muscle injury repair [18]. Early rehabilitation included manual therapy with cross friction massage and myofascial chain release techniques distant from the injury site; pulsed ultrasound therapy (1:4 ratio duty cycle 1 MHz 1 W/cm2 10 minutes); low-level laser therapy (3B 904 nm GaAs laser 2J/cm2, >5 and <60 mW/cm2, 8 minutes); pain-free range of motion (ROM) exercises in all lower limb joints; strengthening program respecting the isometric - concentric - eccentric order; and blood flow restriction (BFR) therapy (60% limb occlusion pressure, four sets of repetitions 30/15/15/15).

We included proprioceptive training from the start of treatment, initially without load on the affected side. Optimal loading was progressed in terms of magnitude, direction, and rate [19], adding external resistance as tolerated, protecting the healing muscle while exposing it to progressive fascicle lengthening challenges [20].

As we did not have access to an anti-gravity treadmill, we instead introduced aquatic therapy in week 6. Initially, the athlete was submerged up to the clavicle level, and, with time, the water level decreased. It is known that the choice of water immersion depth determines the axial load on submerged structures [21]. By gradually decreasing the depth of immersion, we increased weight-bearing in a controlled manner. We were able to introduce functional movement patterns and neuromuscular training without full loading while maintaining the athlete’s physical condition.

After the athlete met all the relevant clinical, radiological, and functional criteria (i.e., no pain, full ROM in all joints, good neuromuscular control with no compensations, and free weight squat at 1.5x body weight), we were able to introduce lower-level plyometric drills at week 10 after injury. We gradually increased plyometric intensity starting with two-legged drills. The athlete started jogging at 11 weeks (5.5-6 km/h) and low-speed straight running at 12 weeks (10.9-11.4 km/h) after the injury, with speed gradually increasing. Following the progression of intensity in plyometric work and running speed, we introduced football-specific work on the field while respecting the control-chaos continuum (CCC). The CCC proposed by Taberner et al. is an RTP framework that interlinks global positioning system (GPS) variables and moves from high control to high chaos situations, progressively increasing running load demands under progressively riskier conditions [22].

After completing 16 weeks of the rehabilitation program and meeting all the functional criteria established by the health and performance department, the athlete returned to training (RTT). The criteria included the absence of clinical symptoms during activities; ultrasound imaging showing features of muscle repair and healing with no signs of acute injury; manual handheld dynamometry for maximal voluntary isometric contraction for knee flexion at 120° and knee extension at 90° limb symmetry index >90%; Illinois Agility Test with an excellent score (<15,2 seconds); psychological readiness; and adherence to the CCC throughout the entire process [3,22,23].

Following a discussion with the coaching staff, the athlete was integrated back into modified training. First, we maintained contact avoidance to ensure safe player interaction, initially placing the athlete in controlled and closed situations, and then progressing to open-field situations. After five weeks of training with the team, the athlete reached >90% of his pre-injury-level GPS metrics, including total distance, maximum speed, accelerations, decelerations, high-speed running, and sprint distance [3,22,24,25].

**Figure 2:**
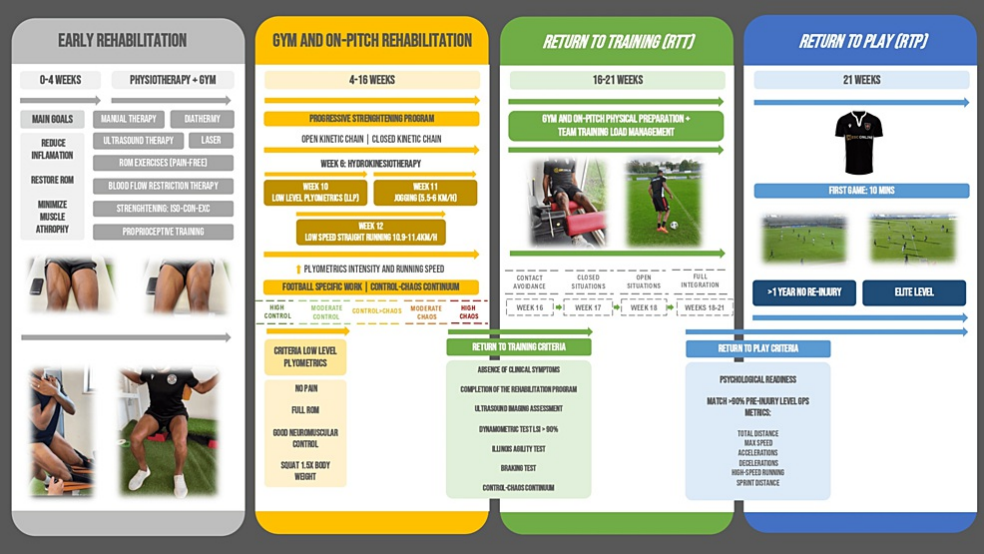
Return-to-play infographic

We considered the athlete physically and psychologically ready for RTP, 21 weeks after his injury. To date, the criteria for RTT and RTP are not evidence-based, given the widespread lack of consensus on this specific topic, especially at the elite level [24]. Therefore, we involved the athlete in RTP decision-making, ensuring he was pivotal in the process (Figure 2).

In his first game after RTP, the athlete played 10 minutes. In his third game, he was in the team’s starting group of 11 and played 65 minutes. One year after RTP, the athlete had no re-injury and is competing at an elite level.

## Discussion

Muscle injuries continue to be a major concern in sports, despite their high incidence and advances in clinical diagnostic criteria and imaging [4]. Optimal management and rehabilitation strategies are still debated in the literature, creating a challenge in decision-making regarding treatment and RTP. Although non-surgical treatment shows a good prognosis in most athletes with muscle injuries, the consequences of treatment failure can be dramatic [4,26].

Considering prognosis indicators of injury severity, it has been reported that high-grade and RF injuries extending into the tendon (BAMIC class c) have a longer RTP time in football players, which may reflect the longer duration required for tendon healing and adaptation in this group [9,27].

Partial-thickness tears of the quadriceps tendon may affect single or multiple layers of the tendon, whereas full-thickness tears involve the rupture of all three layers [28,29]. In this particular case, the athlete sustained a grade 4c muscle injury of the RF according to BAMIC, with complete rupture of the RF distal tendon, which is the superficial layer of the distal quadriceps tendon. Therefore, we also approached the injury as a partial-thickness tear of the quadriceps distal tendon.

According to the most recent literature published, the management of complete proximal RF injuries primarily involves operative treatment [30]. When talking about complete ruptures of distal RF, surgical repair has also been recommended, but there is a lack of evidence to support this recommendation as there are no published studies comparing the functional results and complications between surgical intervention and conservative treatment [10,11,16]. Consequently, there is still no consensus about the management of this particular injury, especially in elite sports, with only a few reports published to date [10,11]. To our knowledge, there is no published clinical case in elite football detailing the RTP process following this kind of injury. In this case, the athlete and the health and performance department chose a nonoperative treatment because of a lack of functional deficit.

Any complete muscle injury is a challenging lesion and carries the least favorable prognosis [11,16,31]. Non-surgical repair of complete muscle injuries is slow and often results in fibrosis, which can lead to incomplete functional recovery [16]. Given the lack of evidence concerning progression in the rehabilitation program, functional criteria, and testing for RTT and RTP, we set the progression in the RTP process respecting muscle biology, using objective data and clinical reasoning. Risk assessment of every functional mark was discussed within the health and performance department to ensure that informed decisions among multiple professionals were made on phased progression and optimal loading.

## Conclusions

The primary aim of this case report is to show that non-surgical treatment is feasible after a complete distal rupture of the RF tendon in an elite football player. In professional-level sports, there is a gap in the literature regarding the treatment of this particular injury. Therefore, treatment options must be discussed carefully with the athlete while explaining the advantages and limitations of each treatment. Thus, informed decisions can be made until more data are available for clear evidence-based recommendations. The athlete was able to RTP in the same season, 21 weeks after injury, allowing him to compete at an elite level in the Portuguese Football First League with no re-injury >1 year after RTP.
